# Amino Acid Composition, Antioxidant, and Cytoprotective Effect of Blue Mussel (*Mytilus edulis*) Hydrolysate through the Inhibition of Caspase-3 Activation in Oxidative Stress-Mediated Endothelial Cell Injury

**DOI:** 10.3390/md17020135

**Published:** 2019-02-25

**Authors:** Yunok Oh, Chang-Bum Ahn, Ki-Ho Nam, Yeon-Kye Kim, Na Young Yoon, Jae-Young Je

**Affiliations:** 1 Department of Marine-Bio Convergence Science, Pukyong National University, Busan 48547, Korea; si565@daum.net; 2 Division of Food and Nutrition, Chonnam National University, Gwangju 61186, Korea; a321@jnu.ac.kr; 3 Food Safety and Processing Research Division, National Fisheries Research & Development Institute, Busan 4608, Korea; dennis011@korea.kr (K.-H.N.); yeonkyekim@korea.kr (Y.-K.K.); dbssud@korea.kr (N.Y.Y.)

**Keywords:** blue mussel, bioactive peptide, endothelial dysfunction, oxidative stress, caspase-3 activation

## Abstract

Enhanced oxidative stress plays a central role in promoting endothelial dysfunction, leading to the development of atherosclerosis. In this study, we investigated the protective effects of the hydrolysates derived from blue mussel (*Mytilus edulis*) against H_2_O_2_-mediated oxidative injury in human umbilical vein endothelial cells (HUVECs). The blue mussel hydrolysates were prepared by enzymatic hydrolysis with eight proteases, and blue mussel-α-chymotrypsin hydrolysate (BMCH) showed the highest antioxidant activities in DPPH radical scavenging, ABTS^+^ radical scavenging, and ORAC value compared to those of the other hydrolysates. BMCH also inhibited Cu^2+^-mediated low density lipoprotein (LDL) oxidation. Treatment of H_2_O_2_ resulted in the decreased HUVEC viability whereas pre-treatment with BMCH increased HUVEC viability and reduced reactive oxygen species (ROS) generation. BMCH pre-treatment increased cellular antioxidant capacities, including levels of glutathione (GSH), superoxide dismutase (SOD), catalase (CAT), and glutathione peroxidase (GPx) against H_2_O_2_-mediated oxidative stress in HUVECs. Flow cytometry and western blot analysis revealed that BMCH pre-treatment significantly reduced H_2_O_2_-mediated HUVEC apoptosis through inhibition of caspase-3 activation. Real-time-qPCR analysis showed that BMCH down-regulated expression of p53 and caspase-3 genes, as well as decreased the bax/bcl-2 ratio. Taken together, these results indicate that BMCH may be useful as functional food ingredients for protecting endothelial dysfunction or related disease.

## 1. Introduction

Endothelial dysfunction is an early event in the development of atherosclerosis, the major cause of cardiovascular diseases, which is the leading cause of death worldwide [1,2,3]. Atherosclerosis occurs with subsequent endothelial damage caused by a number of risk factors, such as dyslipidemias, hypertension, diabetes mellitus, smoking, and obesity [4,5]. These common risk factors for atherosclerosis are associated with an increased production of reactive oxygen species (ROS) [6,7]. ROS can induce damage in endothelium through direct oxidation of cellular protein, lipid, DNA, and small cellular molecules [8]. In addition, oxidative stress by ROS can also activate cell signaling pathways related to atherosclerosis, which subsequently controls the regulation of gene expression [9]. When the endothelium becomes damaged, atherosclerosis plaques filled with cellular debris and form cells begin to accumulate in artery walls, thus resulting in a narrowing of arteries that can subsequently cause a blockage or stroke [10,11]. Usually, there are no signs and symptoms until severe narrowing or total blockage of arteries. Thus, pre-treatment with well-established antioxidants is a good strategy for the preventing of atherosclerosis before incidence of severe atherosclerosis.

Over several decades, marine natural products have been vastly explored due to their excellent antioxidant activity in nutrients, pharmaceuticals, and cosmeceutical industries [12,13,14]. One of the well-characterized methods for preparation of antioxidant peptides from food proteins is enzymatic hydrolysis [15,16,17]. Blue mussel (*M. edulis*) is widely cultured in Korea and used as a foodstuff. Because of rich protein content in blue mussels, antioxidant hydrolysates and peptides from blue mussels by enzymatic hydrolysis were reported by different research groups [18,19]. However, no information is available with reference to an anti-atherosclerotic effect of blue mussel proteins or its hydrolysates against oxidative stress-mediated endothelial dysfunction. In the present study, we prepared eight kinds of blue mussel hydrolysates (BMHs) in order to find suitable anti-atherosclerotic hydrolysates and evaluated a molecular mechanism underlying anti-atherosclerotic effect in oxidative stress-mediated endothelial cell injury. In addition, several marine-derived compounds that are capable of HUVEC protection against oxidative stress have been reported [20,21,22]. Here, we investigated the protective effect of BMH on H_2_O_2_-mediated HUVEC injury. Our finding demonstrated that BMH has a protective effect against H_2_O_2_-induced HUVEC apoptosis associated with the inhibition of an apoptosis related pathway.

## 2. Results

### 2.1. Antioxidant and Inhibitory Effect of BMHs on LDL Oxidation

**Figure 1:**
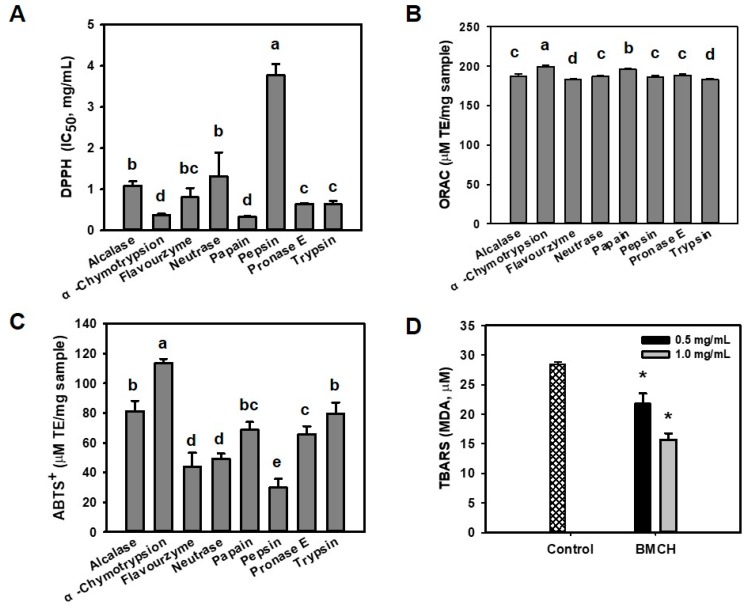
Antioxidant abilities of blue mussel hydrolysates with various proteolytic enzymes determined by (**A**) DPPH scavenging assay, (**B**) ORAC assay, (**C**) ABTS^+^ radical assay, and (**D**) TBARS assay. ^a–d^ Different letters on the bar charts indicated significant differences (*p* < 0.05). * *p* < 0.05 vs. control.

Oxidative stress can promote endothelial dysfunction, which is an initial step of the pathogenesis of atherosclerosis [23]. Prior to evaluate cytoprotective effect of BMHs against oxidative stress-mediated endothelial cell injury, antioxidant activity of BMHs was determined using DPPH, ABTS^+^, and ORAC assays in order to select potential cytoprotective hydrolysate. As shown in Figure 1, papain hydrolysate showed the highest DPPH radical scavenging activity (IC_50_ of 0.33 ± 0.01 mg/mL), followed by α-chymotrypsin hydrolysate (IC_50_ of 0.35 ± 0.03 mg/mL), with no significant difference. Otherwise, α-chymotrypsin hydrolysate showed the highest ABTS^+^ radical scavenging (117 ± 3.5 μM TE/mg sample) and ORAC (199.62 ± 1.42 μM TE/mg sample). Thus, we selected BMH by α-chymotrypsin (BMCH) for further experiments.

LDL is sensitive against ROS and oxidized LDL can promote atherosclerosis, perhaps serving as evidence of atherosclerosis in the early stage [24]. Thus, we evaluated the inhibitory effect of BMCH against Cu^2+^-mediated LDL oxidation by measuring TBARS that were expressed as MDA concentration. Incubation with Cu^2+^ (control) resulted in the significant increase of TBARS; however, this value was decreased by 22.03 ± 1.24 and 15.56 ± 0.65 μM MDA in the presence of BMCH of 0.5 and 1.0 mg/mL. This result indicated that BMCH could inhibit LDL oxidation.

### 2.2. The Protective Effect of BMCH against H_2_O_2_-Induced Cytotoxicity in HUVEC

**Figure 2:**
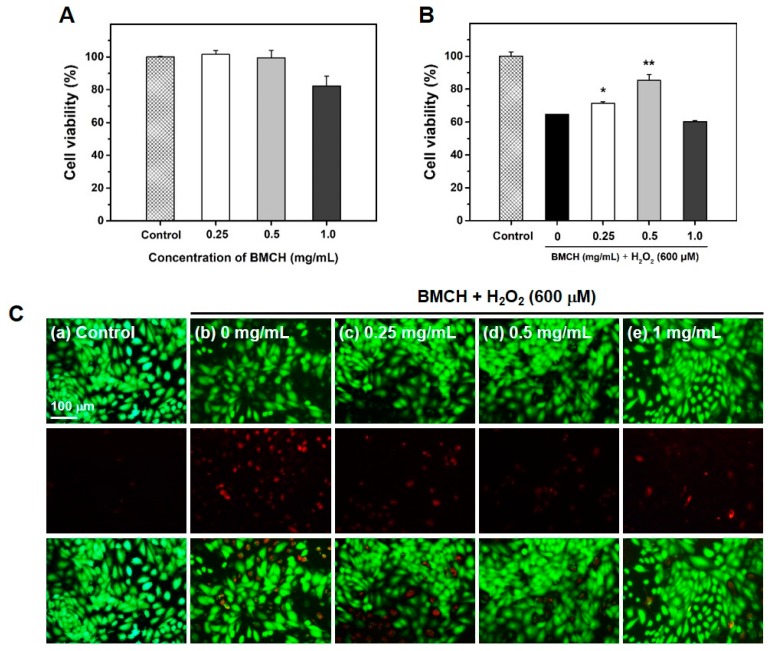
Effect of BMCH on oxidative stress-mediated HUVEC injury. (**A**) Cell viability and (**B**) the protective effect of BMCH in H_2_O_2_-mediated HUVEC injury, as determined by MTT assay, and (**C**) live-dead cell assay by calcein-AM/PI double staining. Cells were pretreated with BMCH for 30 min followed by exposure of H_2_O_2_ (600 μM) and incubation for 24 h. * *p* < 0.05 and ** *p* < 0.01 vs. H_2_O_2_ only treatment.

ROS-mediated vascular endothelial cell damage is strongly related to developing cardiovascular diseases including atherosclerosis [25]. Thus, we established the H_2_O_2_-induced HUVEC injury model and explored whether BMCH could mitigate H_2_O_2_-induced HUVEC injury. The result of cytotoxic assay revealed that BMCH treatment showed no cytotoxic effect up to 0.5 mg/mL; however, BMCH showed around 20% cytotoxic effect on HUVECs at 1.0 mg/mL (Figure 2A). Figure 2B shows the protective effect of BMCH against H_2_O_2_-induced HUVEC injury. A 600 μM H_2_O_2_ treatment significantly decreased HUVEC viability (64.76 ± 0.08%) ; however, pretreatment of BMCH increased HUVEC viability up to 85.35 ± 3.42% at 0.5 mg/mL, indicating BMCH ameliorated H_2_O_2_-induced HUVEC injury.

To further confirm the in vitro protective effect of BMCH in HUVEC injury, live/dead cells observation was carried out using the calcein AM/PI double staining. As shown in Figure 2C, the control (without treatments) clearly showed a strong green fluorescence, indicating a uniformly confluent layer of viable cells. Next, the cells treated with H_2_O_2_ alone displayed a reduced number of viable cells (green) and an increased number of dead cells (red). The cells with pretreatment of BMCH before H_2_O_2_ exposure displayed a reduced number of dead cells with reduced red fluorescence intensity compared to H_2_O_2_ alone treatment, indicating the cytoprotective effect of BMCH against H_2_O_2_-induced HUVEC injury.

### 2.3. BMCH Treatment Inhibits ROS Generation in H_2_O_2_-Induced HUVEC Injury

**Figure 3:**
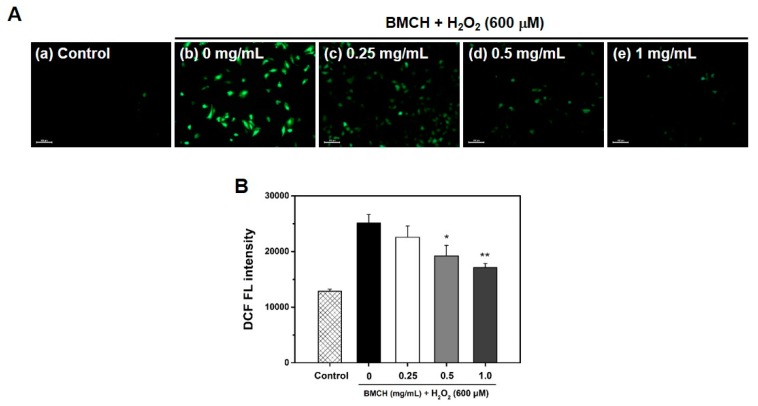
(**A**) Determination of intracellular ROS production by DCFH-DA staining under a fluorescence microscope and (**B**) quantitative intracellular DCF fluorescence intensity in H_2_O_2_-treated HUVEC. Cells were pretreated with BMCH for 30 min, followed by exposure of H_2_O_2_ (600 μM) and incubation for 24 h. * *p* < 0.05 and ** *p* < 0.01 vs. H_2_O_2_ only treatment.

Because ROS is a key regulator in vascular diseases, the inhibitory effect of BMCH on intracellular ROS generation was investigated using DCFH-DA assay. As shown in Figure 3A, H_2_O_2_ alone treatment showed bright DCF green fluorescence, indicating the increase of intracellular ROS generation, whereas the control showed no DCF green fluorescence, and the fluorescence intensity of DCF in the HUVECs treated with BMCH was decreased compared to that of H_2_O_2_ alone treatment. This result indicated that BMCH effectively quenched intracellular ROS in H_2_O_2_-mediated HUVEC injury. We further confirmed by quantification using microplate reader that BMCH treatment decreased the intracellular ROS generation in H_2_O_2_-induced HUVEC injury (Figure 3B). The results indicated that BMCH pretreatment can suppress the intracellular ROS generation from H_2_O_2_-induced HUVEC injury.

### 2.4. BMCH Enhanced the Levels of Intracellular GSH and Antioxidant Enzyme Activities

**Figure 4:**
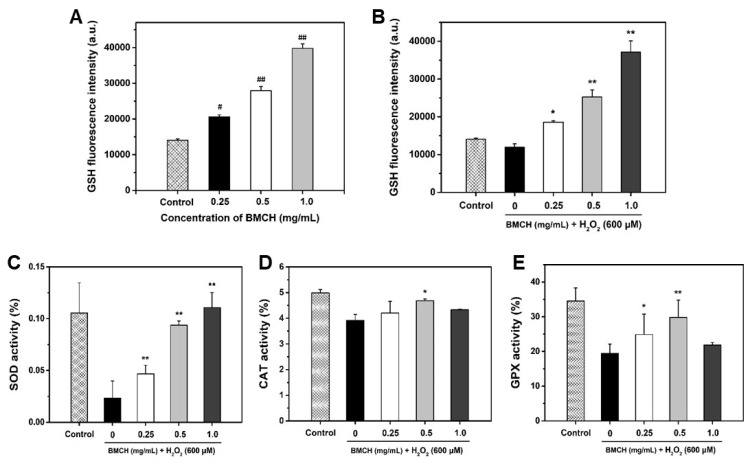
Quantification of intracellular GSH levels under (**A**) normal and (**B**) oxidative stress condition and determination of antioxidant enzyme levels (**C**) SOD, (**D**) CAT, and (**E**) GPx activities. Cells were treated with BMCH for 24 h for measurement of GSH level under normal condition. Cells were pretreated with BMCH for 30 min followed by exposure of H_2_O_2_ (600 μM) and incubation for 24 h for measurement of GSH under oxidative stress condition and antioxidant enzyme activities. Differences were considered statistical significance at ^#^ *p* < 0.05 and ^##^ *p* < 0.01 compared with the control, and * *p* < 0.05 and ** *p* < 0.01 compared with the cells treated with H_2_O_2_ only treatment.

To investigate the protective mechanism of BMCH against H_2_O_2_-mediated HUVEC injury, we first investigated intracellular GSH level. GSH, a well-charaterized antioxidant, is endogeneously synthesized in all cells and plays a key role in antioxidant defense against oxidative stress [26]. As depicted in Figure 4A,B, GSH level was significantly increased by BMCH treatment under both normal and oxidative stress conditions. H_2_O_2_ alone treatment resulted in the decrease of 14.33% in intracellular GSH level compared the control, but reduced GSH level caused by H_2_O_2_ treatment was completely restored and increased by BMCH treatment.

Next, we determined three antioxidant enzyme activities including SOD, CAT, and GPx because these enzymes are associated with cytoprotection against oxidative stress-mediated cell injury. As shown in Figure 4C–E, significant depletions of SOD, CAT, and GPx were detected in H_2_O_2_ treatment compared to that in the control group. However, three antioxidant enzyme activities were significantly restored by BMCH pretreatment.

### 2.5. Prevention of Apoptosis and Necrosis by BMCH Treatment in H_2_O_2_-Mediated HUVEC Injury

**Figure 5:**
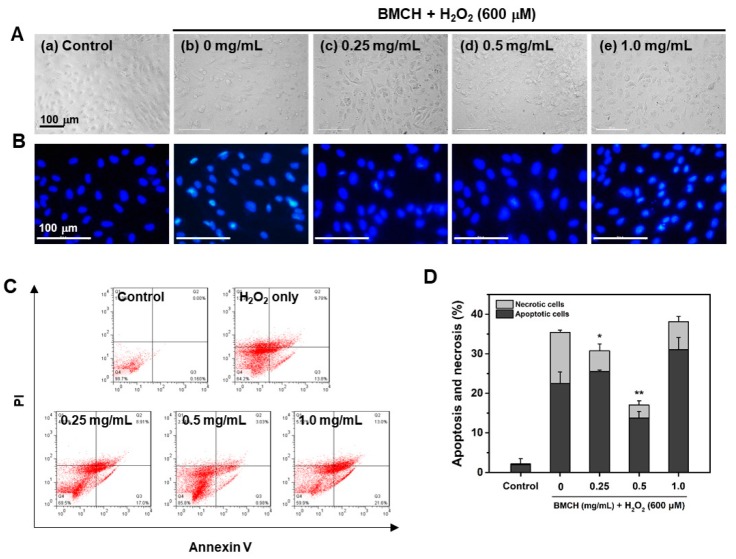
Effects of BMCH on apoptosis in H_2_O_2_-treated HUVEC injury. (**A**) Cellular morphology and (**B**) nuclear morphology by H33342 staining under a fluorescence microscope. (**C**) FACS analysis using Annexin V-FITC apoptosis kit and (**D**) quantification of apoptotic and necrotic cells by FACS analysis. * *p* < 0.05 and ** *p* < 0.01 vs. H_2_O_2_ only treatment.

ROS play an important role in apoptosis induction. Thus, the anti-apoptotic effect of BMCH in oxidative stress-mediated HUVEC injury was determined using Hoechst 33342 nuclear and Annexin V-PI double staining. As shown in Figure 5A,B, injured morphologies with nuclear condensation in the H_2_O_2_ treatment group were observed whereas the control cells were typical HUVEC morphology without nuclear condensation. These morphological changes in H_2_O_2_-mediated HUVEC injury were reversed by BMCH treatment (0.25 and 0.5 mg/mL).

Next, we assessed the effect of BMCH on HUVEC apoptosis and necrosis by H_2_O_2_ treatment using Annexin V-PI double staining (Figure 5C,D). H_2_O_2_ treatment for 24 h increased in the apoptotic (21.41 ± 2.67%) and necrotic cells (13.06 ± 0.95%) compared to the control group, indicating that H_2_O_2_ induced cell death in both pathways. At 0.25 mg/mL of BMCH, necrotic cells were decreased, but apoptotic cells were slightly increased compared to that of the control group. However, total cell death was significantly decreased. At 0.5 mg/mL of BMCH, both necrotic and apoptotic cells were significantly decreased, but treatment of BMCH with high concentration resulted in cell death by increasing apoptosis ; this is in agreement with cytotoxic evaluation in Figure 2. This result indicates that BMCH with low concentration is effective in protection against H_2_O_2_-induced HUVEC injury, but not high a concentration of BMCH in our model.

### 2.6. Modulation of Apoptosis-Related Genes by BMCH in H_2_O_2_-Induced HUVEC Injury

**Figure 6:**
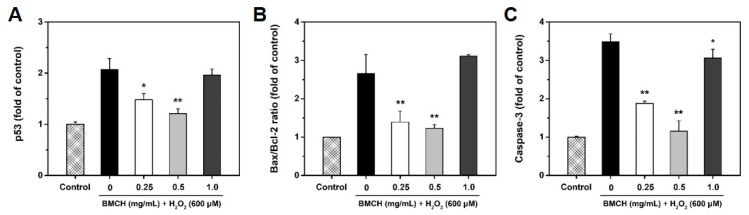
Effect of BMCH on mRNA expressions by RT-qPCR. (**A**) p53, (**B**) bax/bcl-2 ratio, and (**C**) caspase-3. Cells were pretreated with BMCH for 30 min followed by exposure of H_2_O_2_ (600 μM) and incubation for 24 h. * *p* < 0.05 and ** *p* < 0.01 vs. H_2_O_2_ only treatment.

To verify the anti-apoptotic effect of BMCH, we assessed the mRNA expressions of p53, bax, bcl-2, and caspase-3 genes using RT-qPCR. As shown in Figure 6, H_2_O_2_ treatment alone significantly increased the mRNA expression of p53 and caspase-3, and the bax/bcl-2 ratio, reflecting the apoptotic state of cells [27]. The fold increase in p53, caspase-3, and the bax/bcl-2 ratio by H_2_O_2_ treatment alone was 1.07-, 2.48-, and 1.70-fold compared to that of the control group, respectively. However, these increases were decreased by 0.86-, 2.33-, and 1.47-fold in p53, caspase-3, and the bax/bcl-2 ratio by BMCH treatment (0.5 mg/mL) compared to that of H_2_O_2_ treatment alone. But high concentration of BMCH did not reverse the gene expressions. Our results clearly showed that low concentration of BMCH is effective in H_2_O_2_-mediated HUVEC injury through downregulation of apoptotic p53 and caspase-3 genes, and the bax/bcl-2 ratio.

### 2.7. Supression Caspase-3 Activation in H_2_O_2_-Induced HUVEC Injury

**Figure 7:**
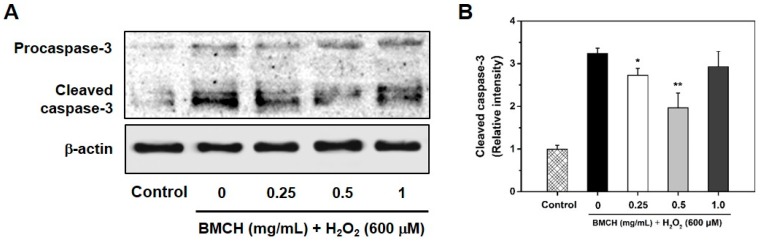
(**A**) Representative western blotting of procaspase-3 and cleaved caspase-3, and (**B**) the relative expression level of cleaved caspase-3 protein level in H_2_O_2_-mediated HUVEC injury. Cells were pretreated with BMCH for 30 min followed by exposure of H_2_O_2_ (600 μM) and incubation for 24 h. * *p* < 0.05 and ** *p* < 0.01 vs. H_2_O_2_ only treatment.

Caspase-3 is an important biomarker of apoptosis associated with ROS generation and activated by a cleavage when an apoptosis event occurs [28]. To investigate further information in correlation with the caspase-3 activation pathway in H_2_O_2_-induced HUVEC apoptosis, caspase-3 expression level was detected by western blotting. As shown in Figure 7, H_2_O_2_ treatment in HUVEC caused the cleavage of procaspase-3 into the cleaved caspase-3, which triggered apoptosis in the cells; however, the cleaved caspase-3 was decreased by BMCH treatment (0.25 and 0.5 mg/mL) but not at high concentration (1.0 mg/mL). The result suggested that BMCH with low concentration supressed H_2_O_2_-induced apoptosis in HUVEC through the inactivation of caspase-3.

### 2.8. Amino Acid Composition and Molecular Weight Distribution of BMCH

BMCH enriched in Glu (9.32%), Asp (7.67%), and Gly (5.98%) (Supplementary Table S1). Molecular weight of BMCH by Q-TOF mass spectrometer ranged from 323.24 to 1138.53 Da (Supplementary Figure S1).

## 3. Discussion

Endothelial cells are directly involved in many functions of vascular biology, including barrier function, vascular homeostasis, inflammation, and formation and repair of blood vessels [29]. Endothelial damage and dysfunction are considered to be an early biomarker in the development of atherosclerosis and a hallmark for vascular diseases [30]. Because oxidative stress is considered to be a major cause in the pathogenesis of atherosclerosis, reduction of oxidative stress toward endothelial cells or LDL oxidation by oxidative stress is a good strategy for the prevention of the pathogenesis of atherosclerosis [31,32,33].

In this study, protein-rich blue mussel was hydrolyzed by eight enzymes to release bioactive peptides with antioxidant and anti-atherosclerotic activity. Antioxidant assays revealed that α-chymotrypsin hydrolysates from blue mussel (BMCH) showed the best antioxidant activity determined by DPPH, ABTS^+^, and ORAC assays, and BMCH also inhibited 53% LDL oxidation mediated by Cu^2+^. Further, we chose H_2_O_2_-induced HUVEC injury as the model for investigation of anti-atherosclerotic effects and the underlying mechanism of BMCH. In this study, H_2_O_2_ treatment resulted in the significant decrease of cell viability with the morphological features of apoptosis. However, BMCH (0.25 and 0.5 mg/mL) treatment could reverse this injury to the normal condition as elevated cell viability, normalized morphology, and reduced apoptosis of HUVECs. These results showed that BMCH treatment may protect against H_2_O_2_-medicated oxidative damage in HUVECs.

ROS plays important roles in the regulation of various functions of cells. The endothelial homeostasis under normal condition can be balanced by the endogenous free radical production/antioxidant defense system, including intracellular antioxidant and antioxidant enzymes such as GSH, SOD, CAT, and GPx. However, overproduction of ROS leads to an imbalance in the oxidant/anti-oxidant mechanisms and subsequently results in oxidative endothelial damage [34,35]. GSH is the most abundant antioxidant in all cells, which can directly scavenge free radicals as an electron donor and act as a substrate for GPx and GST during the reduction of ROS [36]. In addition, SOD catalyzes superoxide radicals to oxygen or H_2_O_2_, followed by the decomposition of H_2_O_2_ through the catalytic reaction of GPx and CAT to water and oxygen. Thus, the activation of these antioxidant capacities is a good therapeutic strategy for the prevention of endothelial dysfunction by oxidative stress. To elucidate the underlying mechanisms of how BMCH treatment reverses this oxidative injury in endothelial cells, we determined cellular antioxidant levels, as well as ROS level in H_2_O_2_-induced HUVEC injury. It is well known that H_2_O_2_ treatment in vascular cells can stimulate ROS production, which are essential regulators of apoptosis, and reduces antioxidant defense capacity [25]. As expected, overproduction of ROS and depletion of antioxidant defense capacity were detected in H_2_O_2_-induced HUVEC injury. On the other hand, BMCH treatment prior to H_2_O_2_ exposure induced enhanced levels of endogenous antioxidant of GSH and antioxidant enzymes of SOD, CAT and GPx compared to that in H_2_O_2_-treated HUVEC alone. These results indicated that BMCH reduced oxidative stress through the elevating of cellular levels of antioxidant molecules.

In addition, oxidative damage induces nuclear damage and stimulates in association with the cascade of apoptotic cell death [37]. Apoptosis is characterized by morphological changes, such as cell shrinkage and chromatic condensation, and specific gene expression related to apoptotic signaling pathways such as p53, caspase-3, and bcl-2 family members [38,39]. p53 is strongly associated with the execution of apoptosis in response to oxidative stress-induced DNA damage [40,41]. In the intrinsic pathway, p53 activation results in an increase of bax and a decrease of bcl-2 expression that cause the release of cytochrome C from the mitochondria. The released cytochrome C activates downstream caspase-9, which subsequently activates caspase-3 that induces apoptosis [42,43]. Our studies clearly showed that BMCH treatment reversed the morphological changes and apoptotic process in H_2_O_2_-treated HUVEC injury. This effect is attributed to the downregulation of apoptotic gene expressions of p53 and caspase-3, and the bax/bcl-2 ratio. However, fold change in p53 and the bax/bcl-2 ratio by BMCH treatment is insufficient to elucidate the anti-apoptotic effect because of a less than 2-fold decrease of p53 gene expression compared to basal and/or H_2_O_2_ treatment alone groups [44]. Moreover, the bax/bcl-2 ratio is an important key factor in the regulation of apoptosis, and a low ratio of the bax/bcl-2 triggers cell death through activating caspase-3 [45]. Although the bax/bcl-2 ratio by H_2_O_2_ treatment alone is insufficiently increased (1.70-fold increase) compared to that of the control group, it could be speculating apoptotic cell death. However, the increased ratio was significantly decreased by BMCH treatment, suggesting that the BMCH treatment probably inhibited the apoptotic process by modulating the bax/bcl-2 ratio. Since the bax/bcl-2 ratio affects the activation of caspase-3, we further confirmed whether BMCH treatment inhibited caspase-3 activation, which is a critical executioner of apoptosis [46]. As expected, the increased bax/bcl-2 ratio by H_2_O_2_ treatment alone up-regulated caspase-3 gene expression (2.48-fold increase), which is further confirmed by western blotting. However, our results clearly showed that BMCH treatment significantly decreased caspase-3 activation in H_2_O_2_-treated HUVEC injury, and this decrease by BMCH treatment may be major protective pathway in our model. Taken together, BMCH treatment as antioxidants inhibits potent apoptotic cell death via enhancing intrinsic cellular tolerance against apoptotic stimuli; thus it can be further explored as potently anti-atherosclerosis.

## 4. Materials and Methods

### 4.1. Materials

Blue mussel (*M. edulis)* was purchased from Yeosu Fisheries Co. (Yeosu, Korea). Hydrogen peroxide (H_2_O_2_), 2,2-diphenyl-1-picryhydrazyl (DPPH), potassium persulfate, 6-hydroxy-2,5,7,7-terramethyl chroman-2-carboxylic acid (Trolox), 2,2′-azino-bis-3-ehtylbenzthiazoline-6-sulfonic acid (ABTS), trifluoroacetic acid (TFA), 2,2′-azobix-2-amidino-proparne dihydrochloride (AAPH), 2,5-diphenyltetrazolium bromide (MTT), monobromobimane (mBBr), 2′7′-dichlorofluorescin diacetate (DCFH-DA), and Hoechst 33342 were purchased from Sigma-Aldrich Chemical Co. (St. Louis, MO, USA). Chloroform, ethanol and dimethyl sulfoxide (DMSO) were purchased from Junsei Chemical Co. (Tokyo, Japan). Phosphate–Buffered Saline (PBS) and Dulbecco’s Phosphate- Buffered Saline (DPBS) were purchased from Hyclone (Logan, UT, USA). The complementary DNA synthesis kit (ET21025) and QuantiSpeed SYBR No-Rox kit (QS105-05) were obtained from PhileKorea (Seoul, Korea). All commercial chemicals were used without further purification.

### 4.2. Preparation of Blue Mussel Hydrolysates

Protein content in blue mussel *M. edulis* was determined by Kjeldahl method [47]. Blue mussel was thoroughly washed using tap water and lyophilized. Lyophilized blue mussel powder was hydrolyzed by eight proteases, including Alcalase (pH 8.0, 50 °C), α-chymotrypsin (pH 8.0, 37 °C), Flavourzyme (pH 7.0, 50 °C), Neutrase (pH 8.0, 50 °C), papain (pH 6.0, 37 °C), pepsin (pH 2.0, 37 °C), Protamex (pH 8.0, 45 °C), and trypsin (pH 8.0, 37 °C), at the enzyme to substrate ratio of 1:100 for 8 h. After inactivation of the enzyme by boiling at 100 °C for 10 min, the hydrolysates were centrifuged at 5000 rpm for 20 min, and the supernatants were freeze-dried. The resultant blue mussel hydrolysates (BMHs) were stored at −20 °C until use.

### 4.3. Determination of Antioxidant Activity

#### 4.3.1. DPPH Radical Scavenging Assay

DPPH radical scavenging activity of hydrolysates was performed as previously described in the methods of Park, Kim, Ahn and Je [19]. Briefly, a 70 μL of BMHs was dissolved in a 70 μL of 150 μM DPPH, and then the mixtures were shaken vigorously for 10 s and kept for 30 min in the dark. The absorbance was measured at 517 nm using a microplate reader after 30 min. The DPPH radical scavenging activity was expressed as IC_50_ value, which is the concentration required to scavenge 50% of the DPPH radical.

#### 4.3.2. ABTS^+^ Radical Scavenging Assay

ABTS^+^ radical scavenging activity was determined by the method of Park and Kim [48]. The stock solution was prepared by mixing with a 7.4 mM ABTS^+^ solution and a 2.6 mM potassium persulfate at a ratio of 1:1. The stock solution was diluted to the working solution with an absorbance of 1.50 ± 0.05 at 414 nm. A 50 µL of BMHs was added in 150 µL of the working solution and incubated for 10 min at room temperature. The absorbance was measured at 414 nm. ABTS^+^ radical scavenging activities were expressed as µM trolox equivalents (TE)/mg hydrolysates.

#### 4.3.3. ORAC Assay

ORAC value was determined according to the method of Zulueta, et al. [49] with slight modifications. Briefly, a 50 µL of BMHs in sodium phosphate buffer (75 mM, pH 7.0) was mixed with 50 µL of fluorescein (78 nM) in a 96-well microplate and incubated at 37 °C for 15 min. After addition of 25 µL of AAPH (221 mM), fluorescence was measured every 5 min for 60 min (Ex/Em = 485 nm/582 nm). ORAC values were expressed as µM Trolox equivalents (TE)/mg hydrolysates.

### 4.4. LDL Oxidation Inhibitory Activity

Inhibitory effect of BMCH on human LDL oxidation by Cu^2+^ was measured by thiobarbituric acid reactive substances (TBARS) assay. Briefly, a 100 µg/mL of human LDL (Lee BioSolutions, Missouri, MO, USA) was oxidized with 10 µM Cu^2+^ at 37 °C for 18 h in the presence or absence of BMCH. After incubation, a 30 µL of EDTA (1 mM) was added to terminate reaction, followed by adding 200 µL of TCA (20% *w/v*) and TBA (1% *w/v* in 0.3% NaOH). The mixture was then heated at 92 °C for 20 min. The mixture was centrifuged at 4000 rpm for 15 min (LZ-1248R, Labogene, Seoul, Korea), and the absorbance of the supernatant was measured at 532 nm. Lipid peroxidation in oxidized-LDL was expressed as µM MDA referring to MDA standard calibration curve.

### 4.5. Cell Culture and Treatments

HUVECs were purchased from the American Type Culture Collection (ATCC; Rockville, MD, USA). The cells were cultured in vascular basal cell medium (ATCC) containing 2% fetal bovine serum (FBS; ATCC), 100 U/mL of penicillin/streptomycin (Hyclone, Logan, UT, USA), and supplemented with endothelial cell growth kit-VEGF (ATCC). The cells were maintained at 37 °C in a humidified incubator with 5% CO_2_, and cell growth medium was changed every two days. The cells were subcultured using a 0.025% trypsin-EDTA solution. The cells used in this study were designated as passage 3 to 5.

To investigate the protective effect of BMCH against oxidative stress, HUVECs were seeded in a 96-well or 6-well plate or 100 mm^2^ dishes and maintained in a 5% CO_2_ incubator. When HUVECs reached 80–90% confluence, the HUVECs were pretreated with BMCH (0, 0.25, 0.5 and 1 mg/mL) for 30 min and then exposed to 600 μM H_2_O_2_ for 24 h. The control group was not treated with BMCH and H_2_O_2_.

### 4.6. Determination of Cytoprotective Effect in H_2_O_2_-Mediated HUVEC Injury

#### 4.6.1. Cell Viability Assay

Cell viability was evaluated by using MTT assay. After treatment with BMCH and H_2_O_2_ in a 96-well plate described above, the MTT solution (0.5 mg/mL) was added to each well and further incubated for 4 h. After removing MTT and adding 100 μL of DMSO, the absorbance of intracellular purple formazan crystals was measured at a wavelength of 540 nm using a GENios microplate reader (GENios, TECAN, Männedorf, Switzerland).

#### 4.6.2. Live/Dead Cell Assay

The live/dead cell assay was utilized for fluorescence staining of viable and dead cells. The cells were simultaneously dual-stained with calcein-AM and PI, which produce green fluorescence in live cells and red fluorescence in dead cells, respectively. After treatment with BMCH and H_2_O_2_ in a 12-well black plate for 24 h, the cells were incubated with 0.5 μM calcein-AM and 5 μM PI dye solution at 37 °C for 20 min while protected from light. After washing PBS, viable and dead cells labeled with calcein-AM and PI were visualized under a fluorescence microscope (Leica DMI 6000 B, Wetzlar, Germany).

#### 4.6.3. Measurement of Intracellular Glutathione (GSH) Levels

GSH levels were evaluated by monobromobimane (mBBr) as a fluorescent thiol probe [50]. After treatment with BMCH and H_2_O_2_ in a 96-well black plate described above, the cells were treated with 40 μM of mBBr for 30 min to form a fluorescent of mBBr-GSH conjugate, followed by detecting fluorescence at the wavelength of Ex/Em 360 nm/465 nm using a GENios microplate reader.

#### 4.6.4. Intracellular ROS Measurement

Intracellular ROS generation was measured using a DCFH-DA in H_2_O_2_-treated HUVECs. After treatment with BMCH and H_2_O_2_ in a 96-well black plate as described above, the cells were incubated with 20 μM DCFH-DA (final Con.) in PBS for 20 min at 37 °C. After washing with PBS, intracellular ROS generation was monitored using fluorescence microscopy, and the DCF fluorescence intensity was quantified at the wavelength of Ex/Em 485 nm/535 nm using a GENios microplate reader.

#### 4.6.5. Determination of Antioxidant Enzyme Activities

After treatment with BMCH and H_2_O_2_ in 100 mm^2^ dishes described above, the cell lysates were prepared using RIPA buffer (Sigma, St. Louis, MO, USA). Cellular antioxidant enzyme activities, including SOD, CAT, and GPx, were determined by using enzyme assay kits (Cayman, Ann Arbor, MI, USA) according to the manufacturer’s instructions.

#### 4.6.6. Cellular Morphological Changes

Hoechst 33342 nuclear staining was conducted to detect apoptotic changes. After treatment with BMCH and H_2_O_2_ in a 6-well plate described above, the cells were fixed with an ice-cold ethanol (75%) for 20 min, followed by staining with 10 μM Hoechst 33342 for 20 min at room temperature. The cells were washed with PBS and the morphology was observed using a fluorescence microscope (Leica DMI 6000 B).

#### 4.6.7. Cell Apoptosis and Necrosis Analysis by Flow Cytometry

Cell apoptosis and necrosis were determined by using an Annexin V-FITC Apoptosis Detection Kit (BD Pharmingen^TM^, San Jose, CA, USA) according to the manufacturer’s protocol. After treatment with BMCH and H_2_O_2_ in HUVECs described above, the cells were harvested and collected at a density of 5 × 10^5^ cells by centrifugation, followed by resuspension in 100 μL of 1× binding buffer. The cells were incubated with 10 μg/mL of Annexin V-FITC/PI double staining solution for 15 min at room temperature in the dark, followed by the addition of 400 μL of 1× binding buffer. The fluorescence was immediately analyzed with flow cytometry (FACSCalibur system, BD Biosciences, San Jose, CA, USA).

#### 4.6.8. mRNA Expression by Real Time-qPCR

Total RNA was isolated using TRIzol reagent (Thermo scientific Co., Waltham, MA, USA). The isolated RNA was converted into cDNA using cDNA synthesis kit (ET21025, PhileKorea, Seoul, Korea). Human p53, caspase-3, bcl-2, and bax transcripts were quantified by RT-qPCR using QuantiSpeed SYBR No-Rox kit (QS105-05, PhileKorea, Seoul, Korea). The PCR reaction was carried out at 95 °C for 2 min to activation of polymerase, followed by 40 cycles of denaturation at 95 °C for 5 s, and annealing/extension at 60 °C for 20 s. Melting temperature was set from 50 °C to 95 °C at 0.3 °C/s. Primers used in this study are listed as follows: β-actin (forward: CTG TCT GGC GGC ACC ACC AT, reverse: GCA ACT AAG TCA TAG TCC GC), bax (forward: TCT GAC GGC AAC TTC AAC TG, reverse: CTC AGC CCA TCT TCT TCC AG), bcl-2 (forward: AGA TGT CCA CFF AGC TGC ACC TGA C, reverse: AGA TAG GCA CCC AGG GTG ATG CAA GCT), caspase-3 (forward: ATT GTG GAA TTG ATG CGT GA, reverse: GGC AGG CCT GAA TAA TGA AA), and p53 (forward: GTT CCG AGA GCT GAA TGA GG, reverse: CTG AGT CAG GCC CTT CTG TC). The β-actin was used a housekeeping gene and all results are normalized to the level of β-actin expression. Relative fold change values were calculated following formula [51]:

Relative mRNA expression = 2^−(ΔC,BMCH − ΔC,blank)^

where, ΔC_x_ = C_x interest gene_ − C_x housekeeping gene_, x is blank or BMCH.

#### 4.6.9. Western Blotting

After treatment with BMCH and H_2_O_2_ for 24 h, protein was isolated using a RIPA buffer (Sigma Chemical Co. St. Louis, MO, USA) and quantified using a Pierce^®^ BCA protein assay kit (Thermo Scientific Co., Waltham, MA, USA). Equal amounts of proteins were loaded and separated by 10% SDS-PAGE gel through gel electrophoresis, and then the proteins were transferred to a PVDF membrane. After blocking with TBS-T buffer containing 5% skim milk, the membrane was incubated with caspase-3 (Cat. No. sc-271759) and β-actin (Cat. No. sc-47778) primary antibodies overnight at 4 °C. After washing with TBS-T, the membrane was further incubated with horseradish peroxidase-conjugated secondary antibody for 2 h. The protein band was imaged on Davinch-Chemi^TM^ imaging system (Core Bio, Seoul, Korea) and the relative intensity of activated caspase-3 protein expression was calculated using imageJ software (version 1.52a, NIH, Bethesda, MD, USA), compared to the β-actin band.

### 4.7. Characterization of Blue Mussel Hydrolysates

The amino acid composition was analyzed by an amino acid autoanalyzer (S43000, Sykam Eresing, Germany) using a cation separation column (LCA K06/Na, 4.6 × 150 mm). Molecular weight distribution was determined using Ultra High Resolution Q-TOF Mass Spectrometer (Bruker Daltonics, Leipzig, Germany).

### 4.8. Statistical Analysis

Data are presented as means  ±  SD. Student’s t-test was used to determine any statistical significance, and the experiments for each sample were repeated at least three times. Differences were considered statistical significant at * *p* < 0.05 and ** *p* < 0.01 compared with the cells treated with H_2_O_2_ only.

## 5. Conclusions

In this study, we demonstrated the anti-atherosclerotic effect of blue mussel hydrolysates by α-chymotrypsin (BMCH). The antioxidant effect by BMCH was attributed to the promotion of intracellular antioxidant defense capacity, such as GSH, SOD, CAT, and GPx, and the inhibition of apoptotic pathway such as downregulation of apoptotic gene expressions of p53, caspase-3, and the bax and upregulation of bcl-2 in H_2_O_2_-induced HUVEC injury. Finally, BMCH treatment represented excellent antioxidant effects leading to the suppression of endothelial cell apoptosis through the casase-3 activation pathway against H_2_O_2_-induced HUVEC injury. Our findings provide new insight of the potential use of marine proteins or hydrolysates as functional food ingredients for protecting endothelial dysfunction.

## Supplementary Materials

The following are available online at https://www.mdpi.com/1660-3397/17/2/135/s1, Figure S1: Molecular weight distribution of BMCH, Table S1: Total amino acid composition of BMCH (g/100 g).
