# Supplementary material for: Amino Acid Composition, Antioxidant, and Cytoprotective Effect of Blue Mussel (Mytilus edulis) Hydrolysate through the Inhibition of Caspase-3 Activation in Oxidative Stress-Mediated Endothelial Cell Injury
Source: Mar Drugs. 2019 Feb 25;17(2):135. doi: 10.3390/md17020135 (PMC6409750; doi:10.3390/md17020135)
Supplement: Supplementary file 1 [file marinedrugs-17-00135-s001.pdf]

marine drugs

Supplementary Materials

Amino Acid Composition, Antioxidant, and Cytoprotective Effect of Blue Mussel (*Mytilus edulis*) Hydrolysate through the Inhibition of Caspase-3 Activation in Oxidative Stress-Mediated Endothelial Cell Injury

Yunok Oh [1,†], Chang-Bum Ahn [2,†], Ki-Ho Nam [3], Yeon-Kye Kim [3], Na Young Yoon [3] and Jae-Young Je [1,*]

1 Department of Marine-Bio Convergence Science, Pukyong National University, Busan 48547, Korea; si565@daum.net
2 Division of Food and Nutrition, Chonnam National University, Gwangju 61186, Korea; a321@jnu.ac.kr
3 Food Safety and Processing Research Division, National Fisheries Research & Development Institute, Busan 4608, Korea; dennis011@korea.kr (K.-H.N.); yeonkyekim@korea.kr (Y.-K.K.); dbssud@korea.kr (N.Y.Y.)
* Correspondence: jjy1915@pknu.ac.kr; Tel.: +82-51-629-6871; Fax: +82-51-629-6865
† These authors equally contributed to this work.

Table 1. Total amino acids composition of BMCH (g/100g).

Amino Acids	g/100g
Asp	7.67 ± 0.18
Thr	3.86 ± 0.18
Ser	2.94 ± 0.13
Glu	9.32 ± 0.19
Pro	2.66 ± 0.25
Gly	5.98 ± 0.27
Ala	3.34 ± 0.19
Cys	0.34 ± 0.19
Val	3.35 ± 0.22
Met	1.65 ± 0.26
Ile	2.99 ± 0.20
Leu	3.42 ± 0.23
Tyr	2.54 ± 0.13
Phe	2.24 ± 0.15
His	2.13 ± 0.13
Lys	3.92 ± 0.22
Arg	4.56 ± 0.22
Total	62.91± 3.16

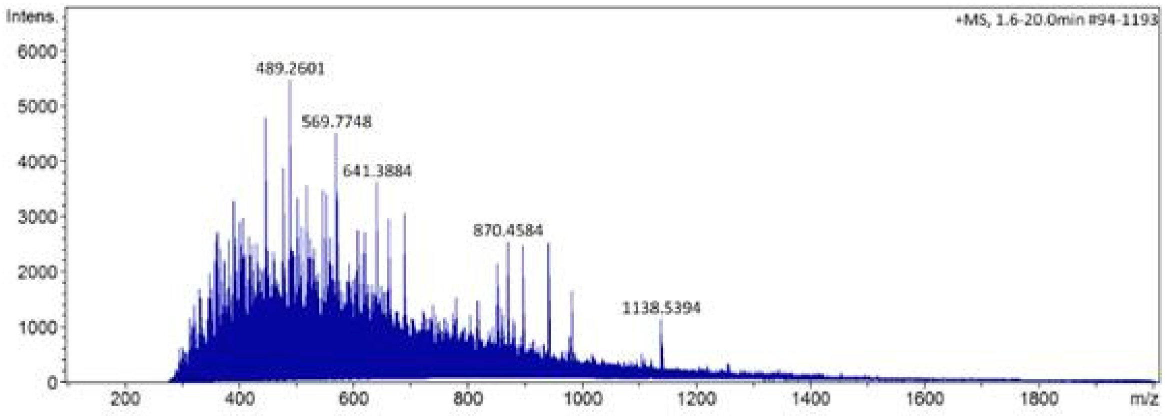

Figure S1. Molecular weight distribution of BMCH.
